# The impact of a faculty development program, the Leadership in Academic Medicine Program (LAMP), on self-efficacy, academic promotion and institutional retention

**DOI:** 10.1186/s12909-021-02899-y

**Published:** 2021-09-02

**Authors:** Judy Tung, Musarrat Nahid, Mangala Rajan, Lia Logio

**Affiliations:** 1 Division of General Internal Medicine, Department of Medicine, Weill Cornell Medicine, 505 East 70th Street HT408, New York, NY 10021 USA; 2 Division of General Internal Medicine, Department of Medicine, Weill Cornell Medicine, 420 East 70th Street LH332, New York, NY 10021 USA; 3 Division of General Internal Medicine, Department of Medicine, Weill Cornell Medicine, 420 East 70th Street LH348, New York, NY 10021 USA; 4 Department of Medicine, Case Western Reserve University School of Medicine, 10900 Euclid Avenue, Cleveland, OH 44106 USA

**Keywords:** Faculty development, Promotion and retention, Course evaluation, Faculty satisfaction

## Abstract

**Background:**

Academic medical centers invest considerably in faculty development efforts to support the career success and promotion of their faculty, and to minimize faculty attrition. This study evaluated the impact of a faculty development program called the Leadership in Academic Medicine Program (LAMP) on participants’ (1) self-ratings of efficacy, (2) promotion in academic rank, and (3) institutional retention.

**Method:**

Participants from the 2013–2020 LAMP cohorts were surveyed pre and post program to assess their level of agreement with statements that spanned domains of self-awareness, self-efficacy, satisfaction with work and work environment. Pre and post responses were compared using McNemar’s tests. Changes in scores across gender were compared using Wilcoxon Rank Sum/Mann-Whitney tests.

LAMP participants were matched to nonparticipant controls by gender, rank, department, and time of hire to compare promotions in academic rank and departures from the organization. Kaplan Meier curves and Cox proportional hazards models were used to examine differences.

**Results:**

There were significant improvements in almost all self-ratings on program surveys (*p* < 0.05). Greatest improvements were seen in “understand the promotions process” (36% vs. 94%), “comfortable negotiating” (35% vs. 74%), and “time management” (55% vs. 92%). There were no statistically significant differences in improvements by gender, however women faculty rated themselves lower on all pre-program items compared to men.

There was significant difference found in time-to-next promotion (*p* = 0.003) between LAMP participants and controls. Kaplan-Meier analysis demonstrated that LAMP faculty achieved next promotion more often and faster than controls. Cox-proportional-hazards analyses found that LAMP faculty were 61% more likely to be promoted than controls (hazard ratio [HR] 1.61, 95% confidence interval [CI] 1.16–2.23, *p*-value = 0.004).

There was significant difference found in time-to-departure (*p* < 0.0001) with LAMP faculty retained more often and for longer periods. LAMP faculty were 77% less likely to leave compared to controls (HR 0.23, 95% CI 0.16–0.34, *p* < 0.0001).

**Conclusions:**

LAMP is an effective faculty development program as measured subjectively by participant self-ratings and objectively through comparative improvements in academic promotions and institutional retention.

## Background

The faculty are the lifeblood of academic medical centers and enabling their success is central to its mission. Multiple factors contribute to a faculty member’s sense of career success, but self-efficacy and work enjoyment are fundamental. Faculty retention and academic promotions are additional measurements of academic success for organizations as well as for individuals. But achievement of faculty satisfaction, promotions and retention are not without challenges.

Only two thirds of medical faculty indicate satisfaction with their current careers [1–3], and almost one third consider leaving their medical college within the next 1–2 years [2, 4]. Faculty attrition, estimated at a rate of 5–8% annually [5–7], is often indicative of discontent and even burnout, which has negative effects on morale [1]. For organizations faculty turnover is also costly, estimated at $400K [5] or twice the annual salary [6] of the departed faculty member. Faculty attrition is disproportionately high in several faculty groups including early career, non-white, female and clinical faculty [5, 7–11] which is problematic at a time when we need to diversify our workforce.

Academic promotion is also a struggle for many faculty and dissatisfaction with the clarity and reasonableness of promotions criteria is high [2, 12, 13]. Only one third of faculty achieve promotions from the assistant to the associate rank within 10 years and women, non-white and clinical faculty are less likely to be promoted [14, 15]. For women, a persistent gap exists in the advancement at all ranks when compared to men [16–18].

Faculty development programming is one method for addressing faculty satisfaction [19], effectiveness [20–22], academic promotions [23, 24] and retention [25], including amongst women faculty [26]. However, prior studies do not distinguish curricular programs from funding efforts [24] and combine promotion in academic rank with promotion in leadership roles [23], making it difficult to isolate the specific impacts of these interventions. Furthermore, retention outcomes are modest, with10–20% improvements even in nationally acclaimed programs [25, 26], and are of unclear reproducibility in other academic medical centers.

## Methods

### Design and setting

At Weill Cornell Medicine, the Leadership in Academic Medicine Program (LAMP) is a 10-month long faculty development offering designed for early career faculty who serve or plan to serve in leadership roles. It consists of monthly afternoon sessions that cover the fundamentals of self-awareness, self-management, career planning and leadership. Seminars led by topic experts included: team building, the Meyer-Briggs Type Indicator®, demystifying the academic promotions process, financing of academic medicine, time management, presentation and publishing skills, mentorship, negotiation, managing others, feedback, conflict resolution and wellness. LAMP also requires participants to develop a capstone project, aligned with their academic goals, in order to apply the learning from the program, e.g., securing a mentor, negotiating for resources, managing competing priorities and presentation of the work. Finally, LAMP includes three 90-min small group sessions of 2 senior mentors paired with approximately 6 LAMP participants to facilitate capstone progress and group mentorship. Adapted from a similar program at Indiana University School of Medicine, LAMP was piloted at Weill Cornell in 2012 in one Department and became a college wide offering in 2013. Interested faculty submit an application that includes a personal statement articulating their career goals, their curriculum vitae, and a nomination letter from their Division Chief or Department Chair pledging support for the applicant’s full participation in the program. Program participants are competitively selected and, 25–40 faculty have enrolled annually.

The purpose of this study was to evaluate several outcomes of the 2013–2020 LAMP cohorts. First, we compared the self-ratings of the LAMP participants on their level of self-awareness, self-efficacy, and satisfaction with their work and work environment before and after the program. Additionally, we evaluated differences in pre and post program self-ratings across gender. Second, we compared promotion in academic rank of the LAMP participants to matched controls. Finally, we calculated the retention of LAMP participants at Weill Cornell Medicine with matched controls. The study protocol was approved by the Weill Cornell Institutional Review Board.

### Study sample

**Fig. 1:**
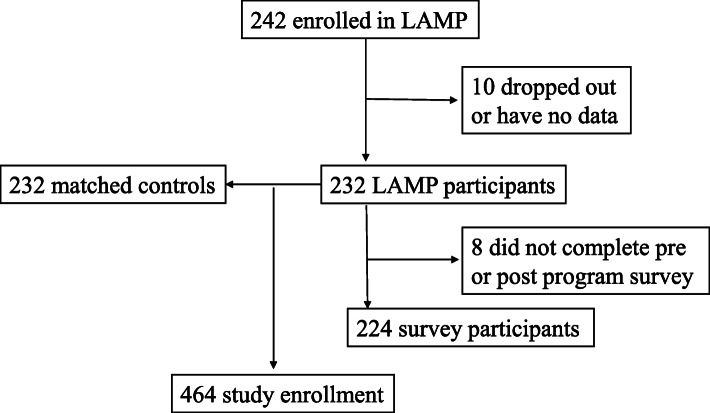
Study enrollment in the Leadership in Academic Medicine Program (LAMP) from 2013 to 2020

Between 2013 and 2020, a total of 242 faculty enrolled in LAMP (Fig. 1). Of those, 10 faculty were excluded due to missing data and/or because they did not complete the program. An additional 232 faculty who had not participated in LAMP were assembled to serve as controls for a total study sample of 464 faculty.

### Program surveys

LAMP participants were asked to complete a pre-program and post-program survey as a stated expectation of the program. The pre-program survey asked about the participant’s academic pathway, area of endeavor (clinical, education, investigation), career concerns and participation goals. The post-program survey solicited feedback on specific components of the program, including evaluation ratings for individual speakers, exercises, and assignments. Both surveys asked the participants to rate, on a 4-point Likert scale, their level of agreement with 14 statements that reflect self-awareness, self-efficacy, satisfaction with work and work environment (Table 2). These items were selected based on our prior knowledge of faculty needs and written to evaluate specific components of our curriculum. The surveys were administered online and at least two reminders were sent to encourage survey completion.

### Matched controls

To assess ascension in academic rank and institutional retention, we assembled a cohort of 464 faculty, 232 LAMP graduates and 232 control faculty. To adjust for confounding effects of gender and environment, we matched each LAMP participant with 1 randomly selected nonparticipant from the institutional faculty database, a repository of all hired faculty. We matched for the following characteristics: (1) gender, (2) initial academic rank, (3) department and (4) hire year. When exact matches were not available, we allowed substitutions for closely related departments and collapsed year of hire into three-year increments. The following departmental combinations were made: (a) Biochemistry with Cell and Developmental Biology; (b) Neuroscience with Brain and Mind Research; (c) Radiation Oncology with Radiology; (d) Microbiology/Immunology and Genetic Medicine were combined with Medicine; and (e) Cardiothoracic surgery, Head and Neck surgery, Neurosurgery and Urology were combined to form Specialty Surgery. This grouping resulted in matches from 18 department groups, down from 26 initial departments. Finally, 6 LAMP participants required individualized assignments: 3 from Rehabilitative Medicine who were matched with controls from Medicine, 2 from Library matched to Oncology and Bioinformatics, and 1 from Surgery matched with Specialty surgery.

### Data analysis

To compare pre and post responses to the survey, McNemar’s tests were done on dichotomized responses (Strongly Agree/Agree versus Strongly Disagree/Disagree). To evaluate differences in responses (original 4-point scale) from pre to post program by gender, we performed Wilcoxon Rank Sum/Mann-Whitney tests.

We constructed Kaplan- Meier curves from time of hire to promotion to next academic rank and to institutional departure for LAMP faculty and matched controls. Group differences were assessed with log-rank tests. Using Cox-proportional-hazards models, we performed two survival analyses: time-to-promotion and time-to-departure. In the time-to-promotion analysis, we did not know when promotion occurred for some subjects, either because they were not promoted during our study period or because they left our organization. The censoring date for these individuals was therefore the date of their departure or the study end date, December 31st, 2020. For the time-to-departure analysis, the survival time of the subject was known if the departure happened within our study period. Data were therefore censored at the end of the study for those still employed.

In recognition of the fact that promotion from the Assistant professor rank to the Associate professor rank is different from promotion from the Instructor rank to the Assistant professor rank, we performed Kaplan-Meier analysis for the subgroup of Assistant Professors.

Analyses were conducted using Stata 15.1 (StataCorp LLC, College Station, TX) and results were considered significant at *p* < 0.05.

## Results

Demographic characteristics of LAMP faculty and matched controls can be found in Table 1. More women faculty (63.8%) participated in LAMP than men (36.2%). The majority (62.1%) were Assistant professors with the remaining (37.5%) entering at the Instructor level; there was 1 Associate archivist accepted into the program as she was still early career. Caucasian race described 53.0% of the LAMP and the control cohort but more LAMP faculty self-identified as ethnic minority, Asian (31.0%), Black (4.3%) and Hispanic (4.3%), than did controls (24.1, 3.0 and 1.7% respectively). LAMP faculty were drawn in largest numbers from the Departments of Medicine, Pediatrics and Anesthesiology. Participants were followed for an average of 5.3 years.

**Table 1:** Subject characteristics: Leadership in Academic Medicine Program (LAMP) 2013–2020

Total sample (*N* = 464)	LAMP faculty *N* = 232		Controls *N* = 232	
Gender				
Female	148	63.8%	148	63.8%
Male	84	36.2%	84	36.2%
Initial Title				
Instructor	87	37.5%	87	37.5%
Assistant	144	62.1%	144	62.1%
Associate	1	0.4%	1	0.4%
Race/Ethnicity				
Caucasian	123	53.0%	122	52.6%
Asian	74	31.0%	56	24.1%
Black	10	4.3%	7	3.0%
Hispanic	10	4.3%	4	1.7%
Other	4	1.7%	4	1.7%
No response	11	4.7%	39	16.8%
Department				
Medicine	85	36.6%	86	37.1%
Pediatrics	26	11.2%	26	11.2%
Anesthesiology	21	9.1%	21	9.1%
Pathology/Laboratory	15	6.5%	15	6.5%
Psychiatry	13	5.6%	13	5.6%
Surgery	9	3.9%	8	3.4%
Obstetrics/Gynecology	7	3.0%	7	3.0%
Radiology	7	3.0%	10	4.3%
Neurology	6	2.6%	6	2.6%
Radiation Oncology	6	2.6%	3	1.3%
Otolaryngology	5	2.2%	4	1.7%
Rehabilitative Medicine	5	2.2%	2	0.9%
Library	4	1.7%	2	0.9%
Ophthalmology	4	1.7%	4	1.7%
Brain and Mind Research	3	1.3%	2	0.9%
Neurosurgery	3	1.3%	3	1.3%
Urology	3	1.3%	2	0.9%
Emergency Medicine	2	0.9%	2	0.9%
Genetic Medicine	2	0.9%	4	1.7%
Population Health Sciences	2	0.9%	2	0.9%
Biochemistry	1	0.4%	1	0.4%
Dermatology	1	0.4%	1	0.4%
Microbiology and Immunology	1	0.4%	3	1.3%
Neuroscience	1	0.4%	2	0.9%

LAMP faculty were matched by gender, academic rank, department, and time period of hire. They were not matched by race/ethnicity

Two hundred twenty-four participants (96.6% response rate) completed both pre and post program surveys. Table 2 shows the changes in the % agreement on 14 statements assessing self-awareness, self-efficacy, satisfaction with work and work environment. There were significant improvements in 11 of 14 items (*p* < 0.05). Greatest improvements were seen in “understand the promotions process” (36% pre-program vs. 94% post-program), “comfortable negotiating” (35% vs. 74%), and “strategy for managing time” (55% vs. 92%). Female faculty rated themselves lower on all items pre-program. The improvements in % agreement was greatest amongst women compared to men in four items: “understand the promotions process”, “comfortable negotiating”, “confident in ability to present work” and “have skills to navigate success”. No statistically significant differences were measured, however, between the improvements in the 4-point scores of women versus men.

**Table 2:** Results of pre and post surveys completed by LAMP participants (*N* = 224

Survey Items (Strongly Disagree, Disagree, Agree, Strongly Agree)	Total (%A, SA)		Female (%A, SA)		Male (%A, SA)	
	Pre	Post	Pre	Post	Pre	Post
1. I recognize my values and priorities	93%	99%	92%	99%	95%	99%
2. I have clearly articulated career goals	73%	96%	71%	95%	79%	96%
3. I feel confident in my ability to progress in my career	78%	92%	76%	91%	81%	95%
4. I have skills in navigating my own success	75%	99%	70%	99%	84%	99%
5. I am comfortable negotiating for what I need to succeed	35%	74%	30%	75%	43%	73%
6. I have a strategy for managing my time and competing demands	55%	92%	54%	91%	57%	93%
7. I am capable of professionally managing conflict in the workplace	73%	93%	70%	92%	77%	95%
8. I am confident in my ability to present my academic work	76%	97%	72%	96%	83%	98%
9. I have an environment of support & guidance for career advancement^	91%	93%	88%	91%	96%	96%
10. I have a mentor who meaningfully contributes to my success	67%	88%	64%	86%	73%	91%
11. I understand the promotions process at Weill Cornell Medical College	36%	94%	31%	96%	44%	91%
12. I look forward to coming to work^	95%	94%	93%	94%	95%	95%
13. I find my work to be personally satisfying^	96%	98%	95%	98%	98%	98%
14. I am satisfied with how my career is advancing	78%	90%	74%	89%	84%	91%

Survey items cluster in these domains: self-awareness (1, 2), self-efficacy (3–8), work environment (9–11), work satisfaction (12–14)

All items on the survey except 3 (^ #9, #12, #13) improved post program, *P* = < 0.05, McNemar’s test

All pre-program items were rated lower by female participants

None of the gender differences were significant by Wilcoxon Rank-sum/Mann-Whitney test

One hundred sixty three (35%) of the study subjects achieved promotion to the next academic rank within the study period, 108 (46.6%) of the LAMP faculty and 55 (23.7%) of the non-LAMP faculty. Cox-proportional-hazards analyses indicate that LAMP faculty were 61% more likely than controls to get promoted (HR 1.61, 95% CI 1.16–2.23, *p* = 0.004).

**Fig. 2:**
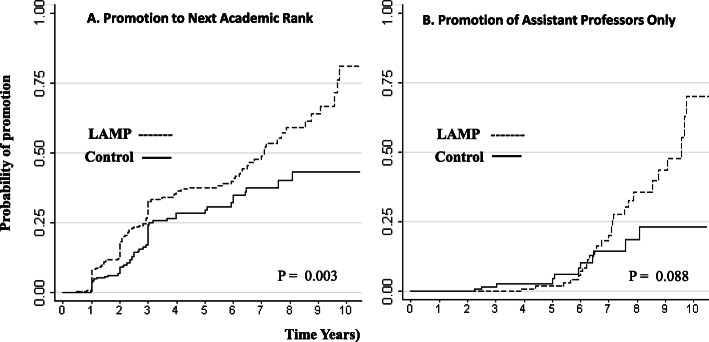
Kaplan-Meier analysis for promotion in academic rank for LAMP faculty and matched controls from year of hire to 2020. **A** Promotion to next academic rank for all study subjects: Instructor to Assistant and Assistant to Associate rank. **B** Promotions for Assistant professors only: Assistant to Associate rank

There was a significant difference found in time-to-next promotion (*p* = 0.003) between LAMP participants and controls. Kaplan-Meier curves reveal that LAMP faculty got to their next promotion more often and faster than controls (Fig. 2A). In the Assistant professor subgroup, there was a trend (*p* = 0.088) toward faster time to promotion to the Associate rank for LAMP participants (Fig. 2B).

**Fig. 3:**
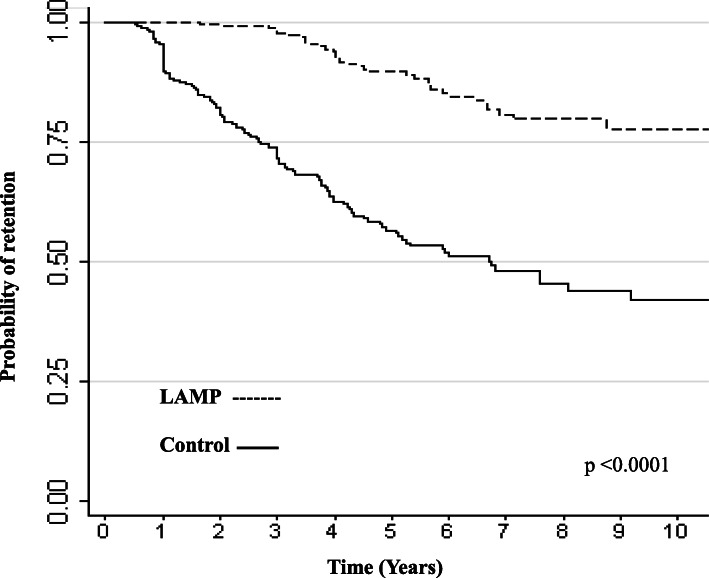
Kaplan-Meier analysis for the institutional retention of LAMP faculty and matched controls from year of hire through 2020

There was a significant difference found in time-to-departure (*p* < 0.0001); LAMP faculty were retained more often and for longer periods than controls (Fig. 3). Overall, 139 (30%) of the subjects left Weill Cornell Medicine, 105 (45.3%) of the non-LAMP controls and 34 (14.7%) of the LAMP faculty. Faculty who participated in LAMP were 77% less likely to leave compared to controls (HR 0.23, 95% CI 0.16–0.34, *p* < 0.0001).

## Discussion

Most faculty development programs evaluate their effect by surveying the satisfaction level of their participants [27]. Few assess for learning, behavior and results, higher levels of impact as defined by the Kirkpatrick model for educational assessment [28]. This study evaluated LAMP for subjective changes in participant ratings as well as objective differences in academic promotion and institutional retention.

We found significant improvements on all domains of self-awareness, self-efficacy, and satisfaction with work and work environment amongst the LAMP graduates. Only 3 out of 14 survey items did not increase significantly, but these scores were high at baseline and had little to no room for improvement. Items that participants rated the lowest at the start of the program (promotions, negotiation, and time management) saw the greatest improvements.

While differences in the score improvements did not significantly differ by gender, it is noteworthy that women self-rated all pre-program items lower than men. Coupled with a higher enrollment of women into LAMP (64%), this suggests that women may desire faculty development more than men and programs directed at women faculty might be needed.

We matched each LAMP participant to a faculty individual of the same gender, hired close to the same year, in the same or similar academic department, and at the same academic rank because these factors contribute to the career trajectory, promotions, and attrition of early career faculty. We achieved good specificity with our matching with preservation of over 18 different departments, although year of hire had to be expanded to a three-year increment.

Comparison to controls revealed that LAMP participants were 61% more likely to achieve promotion to the next academic rank and in a faster duration of time. We chose promotion to the next academic rank as our primary outcome, instead of achievement of a specific rank, because LAMP is designed to support early career faculty, both Instructors and Assistant professors, during a period of career vulnerability [9]. However, in acknowledgement of the fact that ascension to Associate professorship is generally considered more challenging than ascension to Assistant professorship, we performed Kaplan-Meier analysis on the Assistant professors. Subgroup analysis trended towards but did not reach statistical significance possibly due to the smaller number of these faculty or to the longer duration of time needed to get to the Associate level.

Our study’s most significant finding was institutional retention; LAMP faculty were retained more often and for longer periods compared to controls. Only 14.7% of the LAMP faculty left Weill Cornell Medicine during the study period compared to 45.3% of faculty who did not participate in LAMP. National data cite a faculty attrition rate of approximately 7% annually [6–8] which corresponds to the departures in the control cohort. Weill Cornell Medicine offers exit interviews to faculty who leave the organization. Interviews from 119 faculty who departed between 2015 and 2020 reveal that while the majority left for personal or family issues, many cited inadequate support, lack of mentorship and feeling undervalued as part of their reasons for departure. Faculty who participated in LAMP report increased confidence in negotiating support and improved ability to identify and work with mentors, which may contribute to their improved retention. LAMP faculty were observed to be 77% less likely to leave, a remarkable return on investment from an organizational perspective [29]. The greater representation of ethnic minority faculty in the LAMP group is also encouraging when considering diversity in organizational retention efforts, although we cannot draw direct comparisons in retention since we did not match by race and ethnicity. Faculty retention is not actually an explicit goal of LAMP; the program encourages activities and roles that advance the participant’s career even if outside of their home institution.

Finally, LAMP has the potential to impact more than just the faculty who enrolled in the program. Graduates of faculty development programs have been shown to impact their local work environments: transferring knowledge to their peers, fostering collaboration, and leading positive culture change [30]. These important downstream effects can be the focus of future study.

There were several limitations to our study. The dichotomization of survey responses from a 4-point Likert scale might have limited our ability to differentiate degrees of change within items. Promotions in rank is also an incomplete measure of career advancement and we would have liked to have assessed for additional achievements, including grants, awards, publications, and leadership roles. A major limitation of this study was the various confounders that influence retention and promotion that we could not control for. Faculty who seek faculty development opportunities may have inherent differences, including a greater commitment to academic advancement or increased engagement with their organization, compared to faculty who do not participate in faculty development. We do not know if or how often control subjects sought faculty development training or the nature of those courses compared to LAMP. And while we cannot definitively compare the interest or participation level of LAMP faculty to that of control faculty, it is reasonable to assume that individuals who enroll in at least one faculty development program have greater engagement with development efforts. The engagement of the division chiefs and chairs, through the nomination process, could also have influenced the promotion and retention rates of the LAMP faculty. Attention from departmental leaders is expected to accelerate mentorship, promotions, and retention efforts, in fact a goal of the program. In summary, while we cannot conclude causality from LAMP and the observed outcomes, the association between participation in LAMP and improved promotion and retention of its faculty is strong.

## Data Availability

The datasets generated and/or analyzed during the current study are not publicly available due to the local and proprietary nature of the data and because the data contained protected health information (PHI) which is protected under HIPAA regulation, but are available from the corresponding author on reasonable request.
